# Acetabular Fracture and Triradiate Cartilage Injury in an Adolescent After a Motorcycle Crash: A Case Report

**DOI:** 10.7759/cureus.69579

**Published:** 2024-09-17

**Authors:** Sridhar Karne, Jesse J Trent, Tyler C McDonald, Jeffrey L Brewer

**Affiliations:** 1 Department of Orthopedic Surgery, University of South Alabama College of Medicine, Mobile, USA

**Keywords:** cartilage, case, injury, report, triradiate

## Abstract

The acetabular triradiate cartilage is the main structure that determines the development of the acetabulum. Furthermore, the paucity of data in the literature and lack of consensus amongst physicians following diagnosis regarding whether to treat these fractures operatively or non-operatively places the physician with a challenging choice. Here, we report a case of a 13-year-old boy who suffered a triradiate cartilage fracture from a high-energy motorcycle crash and presented with a displaced, left T-type acetabulum fracture. The fracture was successfully treated surgically with open reduction and internal fixation (ORIF). At one year postoperatively, the patient has returned to daily activities, including noncontact sports, with no pain.

## Introduction

The acetabular triradiate cartilage is the main structure that determines the development of the acetabulum. Traumatic injuries to this region are rare in skeletally immature children and account for only 1-2% of all pediatric fractures [1]. Typically, fractures of the triradiate cartilage are seen in children who are involved in a high-energy motor vehicle accident and are associated with soft tissue injury and hemorrhage; associated injuries are often life-threatening [1]. Due to the rarity of these cases, there is a lack of consensus amongst physicians and the literature regarding the most appropriate management of these patients [2]. The goals of operative and non-operative treatment are to restore or maintain anatomy, thus reducing the risk for developmental disturbances such as post-traumatic hip dysplasia [3]. Additionally, with a constellation of associated injuries, the child’s resuscitation status and systemic physiology must be evaluated when determining a treatment plan. As a result, selecting the appropriate treatment can present a dilemma to the treating surgeon. Here, we report a case of triradiate cartilage fracture that was surgically treated with open reduction internal fixation, and the patient is doing well at a one-year follow-up.

## Case presentation

**Figure 1:**
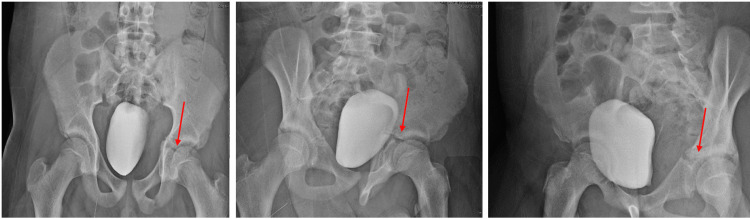
AP and Judet views of left triradiate cartilage fracture.

**Figure 2:**
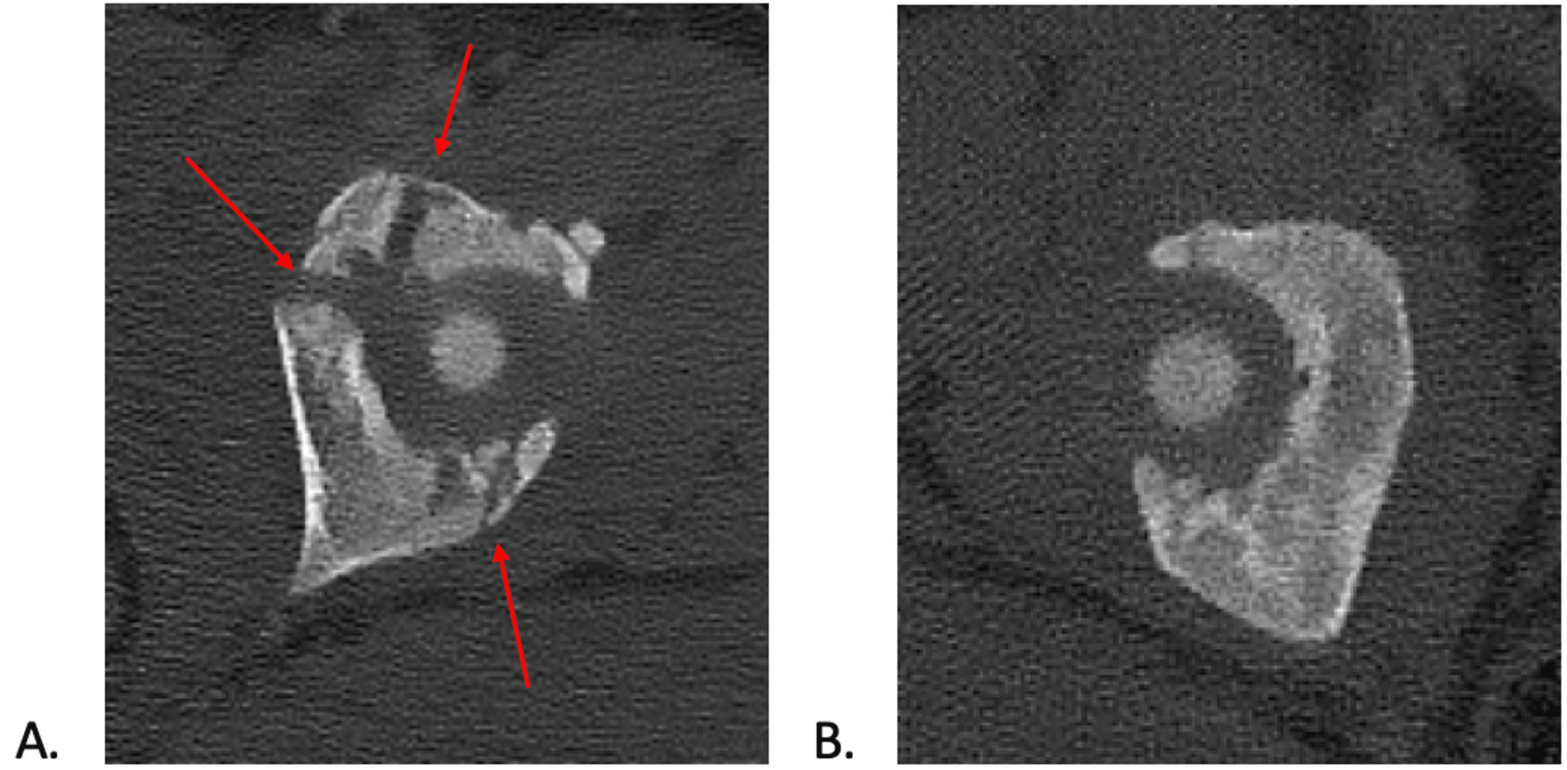
Select axial cuts from computed tomography scan of (a) left triradiate cartilage acetabular fracture and (b) uninjured acetabulum comparison.

**Figure 3:**
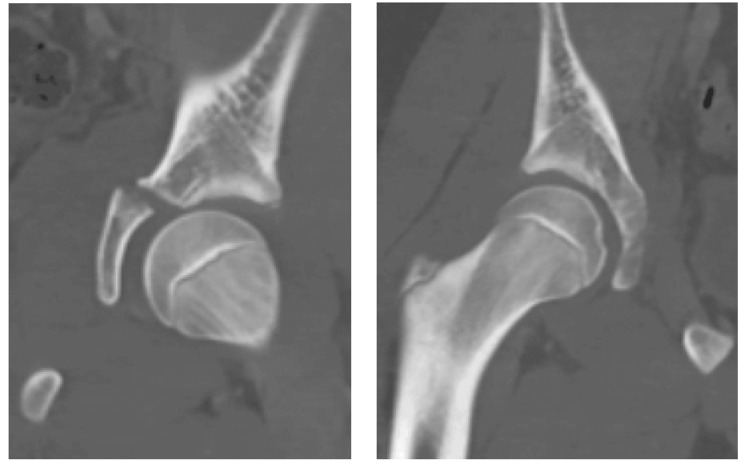
The coronal CT section of left triradiate cartilage acetabular fracture shows nonconcentric positioning of the hip with displacement appreciated.

**Figure 4:**
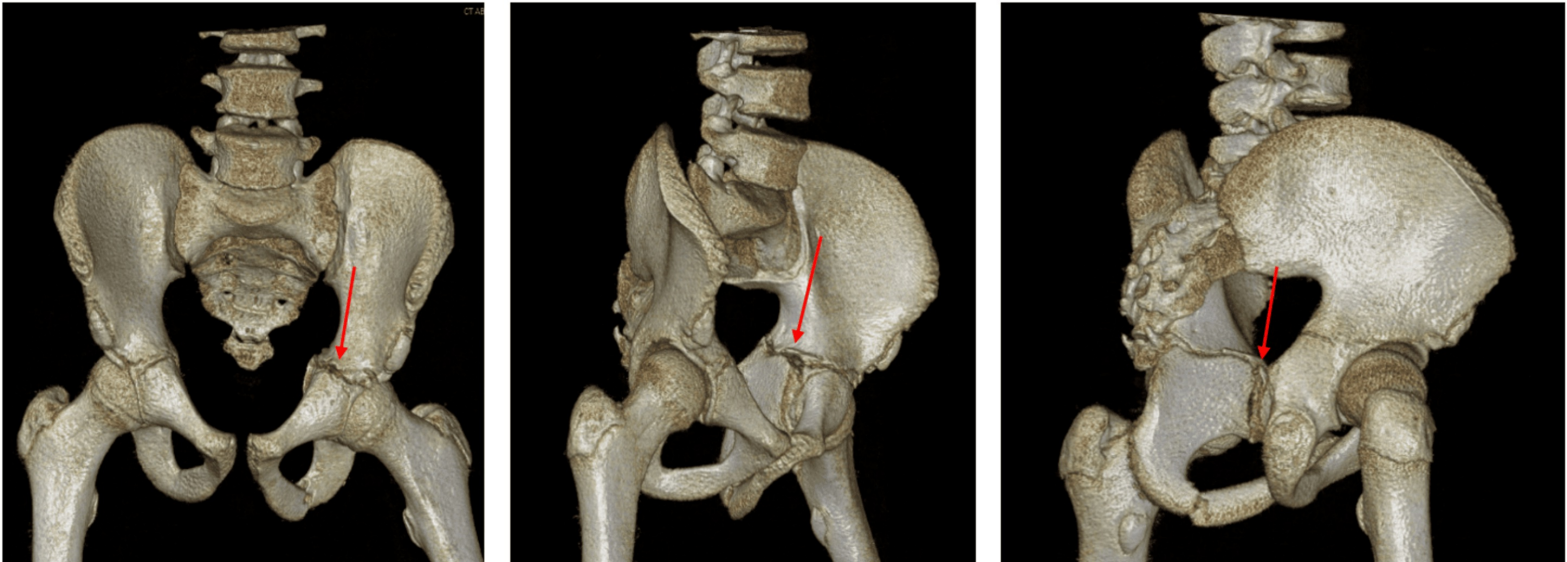
Three-dimensional reconstruction of left triradiate cartilage fracture.

A 13-year-old boy with no significant past medical or surgical history presented to the emergency room following a high-energy motorcycle crash with a displaced, left T-type acetabulum fracture. Concurrently, the patient had a right ankle fracture as well as other non-orthopedic injuries: right traumatic pneumothorax, right 11th rib fracture, right renal laceration, pancreatic laceration, and T7-T9 inferior endplate fractures. Radiographic imaging of the pelvis revealed a left-sided T-type acetabulum fracture classified as a Bucholz-type V cartilage injury of the left acetabulum (Figures 1-4). Considering acetabular joint incongruity, as well as the near full closure of the contralateral acetabulum, ORIF was indicated and performed.

**Figure 5:**
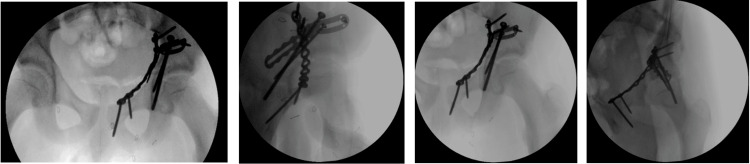
Intraoperative fluoroscopy images.

At a two-week follow-up, the patient had minor peri-incisional numbness on both the middle and lateral windows. Pelvic fluoroscopy images showed that the fracture remained in appropriate alignment (Figure 5).

**Figure 6:**
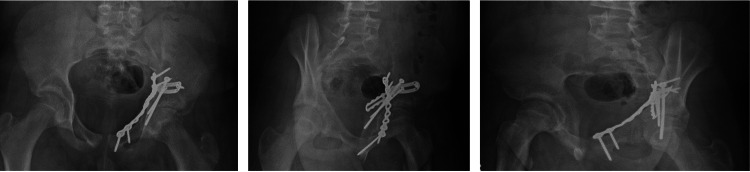
AP and Judet views of pelvis at three-month follow up.

At three-month follow-up, the patient began to walk unassisted without pain. The left inferior pubic ramus fracture healed, and there was no gross pubic symphysis diastasis (Figure 6). The patient was cleared to return to daily activities with no restrictions.

**Figure 7:**
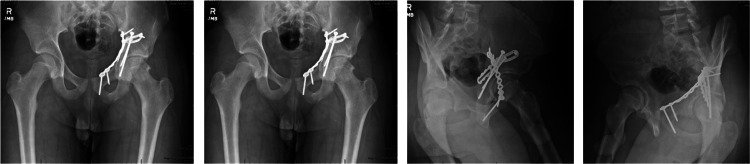
AP and Judet views of pelvis at one-year follow-up.

At a one-year follow-up, the patient is doing well and has returned to sports activities with no pain during daily activities. The left acetabular joint shows some minor joint space narrowing and femoral head osteophytes (Figure 7).

## Discussion

Triradiate cartilage injuries of the acetabulum are often associated with life-threatening injuries and can give rise to growth disturbances in young, skeletally immature patients [4]. Furthermore, complications risks are high from trauma to the triradiate cartilage and blood supply to the hip, causing premature physeal closure. Post-traumatic acetabular injury with subsequent premature physeal closure can lead to complications of femoral head lateralization and subluxation [5]. Previously, conservative management of these fractures in children has been the short-term favored management. This is due to the reassuring capacity of developing children to restore alignment through bone remodeling, making anatomical reduction less essential [6]. However, conservative treatment poses a serious long-term concern of hip dysplasia, chronic pain, and growth disturbances [7]. Still, the physician faces a dilemma due to the rarity of this injury and the paucity of evidence-based treatment. As a result, several factors must be considered when faced with choosing operative versus non-operative management of triradiate cartilage fractures.

The primary evaluation favoring operative management is joint incongruity and instability of the acetabular joint. Most young patients with acetabular injuries present following a high-energy traumatic injury such as a motor vehicle accident and, therefore, are more likely to be polytraumatized [8]. As a result, it is important to consider the overall clinical stability, patient well-being, and level of recovery prior to proceeding with operative management. Second, non-union rates have been established to be lower in adolescent patients [9]. However, in this case presentation, the patient's uninjured right triradiate cartilage appeared closed, indicating that the patient should be treated as a more skeletally mature patient, with more clearly defined indications for operative management.

We report a case of successful operative management of a triradiate cartilage fracture with open reduction internal fixation in a 13-year-old male who, at one year post-operatively, is doing well and has returned to daily sports activities with minimal pain. There are still little data in the literature regarding the management of these cases. Therefore, further research and larger studies are needed to better understand the appropriate management of these patients. Longer follow-up in patients following triradiate cartilage fractures is also needed to investigate long-term outcomes.

## Conclusions

Considerations for the operative versus non-operative management of triradiate cartilage fractures of the acetabulum are unclear and not well understood. The overall stability of the patient, skeletal age, joint incongruity and instability, and risks associated with both types of management should be weighed and carefully considered to determine the most appropriate management of triradiate cartilage acetabular fractures. Close follow-up of these patients following surgery is crucially important to reduce complications and to monitor the progression for possible development of long-term complications such as post-traumatic arthritis and growth disturbances.
